# Nanoporosity of Alumina Surfaces Induces Different Patterns of Activation in Adhering Monocytes/Macrophages

**DOI:** 10.1155/2010/402715

**Published:** 2010-12-28

**Authors:** Natalia Ferraz, Jaan Hong, Matteo Santin, Marjam Karlsson Ott

**Affiliations:** ^1^Division of Surface Biotechnology, Department of Physical and Analytical Chemistry, BMC, Uppsala University, P.O. Box 577, 751 23 Uppsala, Sweden; ^2^Division of Clinical Immunology, Rudbeck Laboratory, Department of Oncology, Radiology and Clinical Immunology, University Hospital, Uppsala University, 751 85 Uppsala, Sweden; ^3^School of Pharmacy and Biomolecular Sciences, University of Brighton, Brighton BN2 4GJ, UK

## Abstract

The present study shows that alumina nanotopography affects monocyte/macrophage behavior. Human mononuclear cells cultured on alumina membranes with pore diameters of 20 and 200 nm were evaluated in terms of cell adhesion, viability, morphology, and release of proinflammatory cytokines. After 24 hours, cell adhesion was assessed by means of light microscopy and cell viability by measuring LDH release. The inflammatory response was evaluated by quantifying interleukin-1*β* and tumour necrosis factor-*α*. Finally, scanning electron microscopy was used to study cell morphology. Results showed pronounced differences in cell number, morphology, and cytokine release depending on the nanoporosity. Few but highly activated cells were found on the 200 nm porous alumina, while relatively larger number of cells were found on the 20 nm porous surface. However, despite their larger number, the cells adhering on the 20 nm surface exhibited reduced pro-inflammatory activity. The data of this paper implies that nanotopography could be exploited for controlling the inflammatory response to implants.

## 1. Introduction

Materials intended for applications in humans, that is, biomaterials in the form of medical devices and prostheses, trigger biological responses such as inflammation, tissue repair, and regeneration when implanted into living tissue [1–3]. The initial event after implantation is the adsorption of plasma proteins onto the material surface, a complex procedure involving dynamic interactions between the proteins and material surface, leading to conformational changes of the proteins [4–7]. The subsequent phenomena, that is, activation of the cascade systems of blood and recruitment and adherence and activation of blood cells, are governed by the initially adsorbed proteins [8, 9]. Platelets, neutrophils, and monocytes adhere to the plasma protein-coated biomaterial surface and become players in the activation of an inflammatory process. In particular, monocyte-derived macrophages play a key role in this immune process. Upon activation, macrophages secrete cytokines, chemokines, growth factors, and other bioactive agents that modulate the function of both immune-competent and tissue cells. Therefore, macrophages have a central role in the tissue repair process, mediating the clearance of tissue debris and bacteria and promoting wound healing through tissue-cell migration and proliferation. In the case of implanted materials, the presence of a layer of denaturated proteins is likely to alter the activity of these cells leading to chronic inflammation and to the development of a fibrotic tissue that prevents the integration of the implant with the surrounding tissue [1, 10].

Biologically inspired materials are being developed with the aim of improving the integration of medical implants and, as a consequence, their clinical performance [11]. Among the various biomimetic approaches, a promising strategy is the design of topographically patterned surfaces that resemble those found in the biological extracellular environment of the tissues [12–14]. Indeed, a broad range of cells (osteoblast, fibroblasts, neutrophils, macrophages, endothelial, epithelial, and smooth muscle cells) have been shown to react to nanoscale features in terms of cell adhesion, morphology, orientation, and activity. These features included grooves, ridges, spikes, islands, wells, nodes, and pores [12, 15–19].

Nanoporous alumina has been recognized as an important material and as a template for the fabrication of nanostructures [20]. Anodic oxidation of aluminium in polyprotic acids produces a well-ordered structure of nanoporous alumina, where pore size can be determined by varying the applied voltage [21]. Previous works have shown that nanoporous alumina has a great influence on both complement activation and platelet adhesion and activation [22–24].

In this paper, the effect of nanopore size on human monocytes/macrophages (MM) in terms of cell adhesion, morphology, and release of proinflammatory cytokines is described. Alumina membranes with two distinct pore sizes (i.e., 20 nm and 200 nm in diameter) were compared for their ability to activate human MM. Alumina membranes were treated with human plasma to induce the formation of an adsorbed protein biofilm and then incubated for 24 hours with freshly isolated human MM. Scanning electron microscopy was used to evaluate the morphology of the adhering cells. Levels of cell adhesion and viability were quantitatively evaluated by means of light microscopy and by measuring lactate dehydrogenase (LDH) activity in the supernatants. Finally, cell activation was also assessed by quantifying the release of proinflammatory cytokines such as interleukin-1*β* (IL-1*β*) and tumour necrosis factor-*α* (TNF-*α*).

## 2. Materials and Methods

### 2.1. Nanoporous Alumina

**Figure 1:**
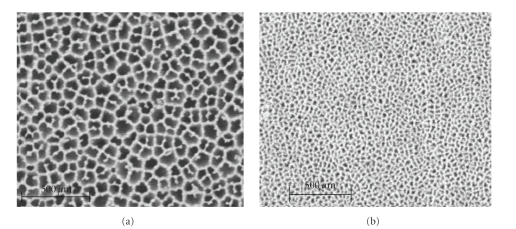
SEM micrographs showing nanoporous alumina membranes with pore diameters of 200 nm (a) and 20 nm (b).

Nanoporous alumina membranes with pore diameters of 20 and 200 nm (Anodisc Whatman International Ltd, Madison, England) were used in this study. The membrane discs were 13 mm in diameter and 60 *μ*m thick with narrow pore size distribution. According to Karlsson et al. [19], the membranes have similar surface roughness and surface chemical characteristics independent of porosity. Readers are referred to the work done by Karlsson et al. [19] for detailed chemical compositions of the nanoporous alumina membranes.  Figure 1 shows representative SEM micrographs of the surfaces of 20 and 200 nm alumina membranes.

### 2.2. Isolation and Culture of Mononuclear Cells

Whole venous blood was freshly collected from seven healthy donors in heparin-coated 50 mL Falcon tubes (Becton Dickinson, USA) containing soluble heparin (Leo Pharma A/S, Ballerup, Denmark), giving a final concentration of 1 IU heparin/mL. Human monocyte enrichment cocktail (RosetteSep, StemCell Technologies, Vancouver, BC, Canada) was added to the heparinized blood according to the manufacturer's instructions. After 20 min of incubation, mononuclear cells were isolated using Ficoll-paque medium (Amersham Biosciences AB, Uppsala, Sweden). Briefly, blood samples containing the enrichment cocktail were layered on to Ficoll-paque medium and then centrifuged for 20 min at 1200× g at room temperature. The mononuclear cell ring formed at the plasma-Ficoll interface was collected and washed three times with Hank's balanced salt solution (HBSS, Invitrogen, UK), containing 2% (v/v) foetal calf serum (FCS). Cells were finally resuspended in RPMI-1640 medium (Sigma, UK), supplemented with 5% (v/v) FCS, 100 IU penicillin/mL, and 100 *μ*g streptomycin/mL, and counted using a haemocytometer. Cell viability was assessed using trypan blue staining (95% cell viability). The cell isolation step provided a fraction of platelet rich plasma (PRP) which was collected and used for coating the materials. Alumina membranes were placed in 24-well tissue culture plates and coated with 1 mL of PRP for 30 min at 37°C, followed by 3 washing steps with HBSS. Cells were added to the wells (4 × 10^5^ cells/well) and cultured for 24 h at 37°C, 5% CO_2_ in a humidified atmosphere. After 3 h of incubation, medium was changed to remove nonadherent lymphocytes. At the end of each experiment, supernatants were collected, centrifuged, aliquoted, and stored at −70°C for future analyses. The nanoporous alumina membranes were fixed with 1.5% (v/v) glutaraldehyde and stored at 4°C until microscopy analyses.

### 2.3. Cell Number: Light Microscopy

Adherent cells were fixed with 1.5% (v/v) glutaraldehyde, stained with crystal violet, and observed under a light microscope (Carl Zeiss, Jena Germany). The density of adherent MM was determined by counting cells in 9 representative 20x objective fields (on duplicate samples) and expressed as number of MM (± standard error) per membrane (*n* = 7).

### 2.4. Cell Viability: LDH Release

Nonviable cells were estimated by analyzing the activity of lactate dehydrogenase (LDH) in the culture medium. Duplicate samples of culture medium were analyzed with a LDH activity kit (In vitro toxicology assay kit LDH based, Sigma, Missouri, USA). The assay measures LDH activity via the reduction of NAD. The resulting reduced product (NADH) is used in the stoichiometric conversion of a tetrazolium dye, and the resulting coloured compound is measured spectrophotometrically. The enzyme activity was measured by reading the absorbance at 492 nm and corrected by the values of the blank (RPMI medium supplemented with 5% (v/v) FCS, 100 IU penicillin/mL, and 100 *μ*g streptomycin/mL). A negative control was done by measuring LDH activity of cells cultured on Thermanox (Thermanox Coverslips, Nalge NUNC International, Rochester, USA). Cultured cells lysed by Triton-X100 served as positive control. LDH release was expressed as arbitrary units (optical density at 492 nm) and normalized by the number of adherent cells. Values were given as mean ± standard error of the normalized LDH release from *n* = 7.

### 2.5. Cytokine Release: IL-1*β* and TNF-*α*

Cytokine production was quantified in duplicate samples of supernatant by enzymatic immunoassays. TNF-*α* and IL-1*β* were assayed by ELISA kits (Human IL-1*β*/IL-1F2 and Human TNF-*α*/TNFSF1A Quantikine HS, R&D systems, UK) following the manufacturer's protocol. Cytokine values were obtained as pg/mL and then normalized by the number of cells adhering on the corresponding membrane. Values were expressed as mean ± standard error from *n* = 7. The number of nonadherent cells was negligible, and their production of cytokines was assumed to be below the kit sensitivity.

### 2.6. Cell Morphology: Scanning Electron Microscopy

After culture, the alumina membranes were fixed with 1.5% (v/v) glutaraldehyde, dehydrated through a series of acetone concentrations (25, 50, 70, 80, 90, and 100% by volume), and critically point dried and sputtered with gold, finally to be studied using an LEO 1530, Gemini SEM. The degrees of plasmalemma roughness and cell spreading were evaluated as indicators of cell activation.

### 2.7. Statistics

Data were statistically evaluated by the Student's *t*-test for unpaired samples, using Statview software for Macintosh. Samples were considered statistically different at either *P* < .001 or  *P* < .05.

## 3. Results

### 3.1. Cell Number

**Figure 2:**
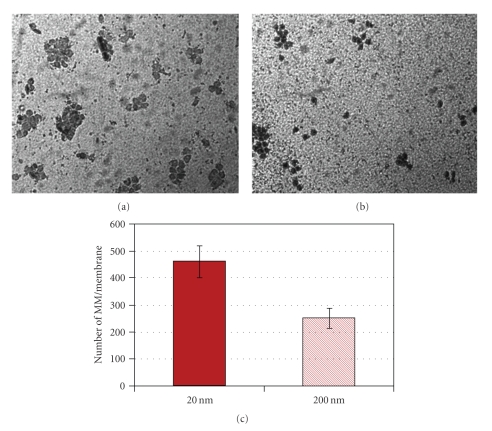
Representative light microscopy images of crystal violet-stained MM on (a) 20 nm-pore alumina and (b) 200 nm-pore alumina. (c) Number of MM on alumina membranes determined by means of light microscopy. Cells were counted in 9 representative 20x objective fields on each membrane. Data represents the mean ± SE from experiments using blood from 7 different donors. Cell adhesion on the 20 nm-pore alumina membrane was significantly higher than on the 200 nm-pore alumina (*P* < .05).

Nanoporous alumina membranes displayed poresize-dependent monocyte/macrophage adhesion. Examination of the nanoporous surfaces after 24 h under light microscopy showed that MM adhesion on the 20 nm-pore alumina membranes was significantly higher than on the 200 nm-pore membranes (Figure 2). This increase was significantly different and approximately 100% higher.

### 3.2. Cell Viability

Measurement of released LDH normalized by the number of adherent cells indicated no significant difference between the studied materials: 20 nm (4 ± 1) 10^−4^ AU/cell number, 200 nm (10 ± 2) 10^−4^ AU/cell number, *P* > .05. Comparison with the negative control indicated similar cell viability: Thermanox (3 ± 1)10^−4^ AU/cell number, *P* > .05. Cells lysed by Triton-X 100 showed 10 times higher values of normalized LDH release (*P* < .05). Hence, the studied material showed no significant toxicity, and cell viability was comparable with a commonly used tissue culture substrate (Thermanox).

### 3.3. Cytokine Release

**Figure 3:**
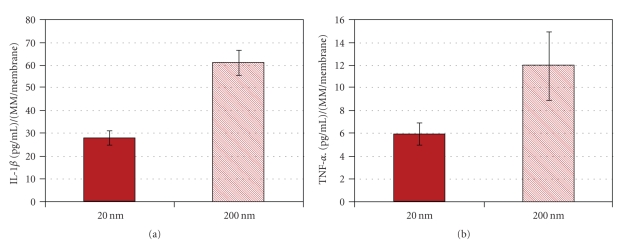
Cytokines released by MM after 24 h of incubation with 20 nm- and 200 nm-pore alumina. Data represents the mean ± SE from experiments using blood from 7 different donors. A significant difference between the two materials was observed for IL-1*β* release (*P* < .001), as well as a clear tendency for TNF-*α*.

Comparison of IL-1*β* secreted by cells attached to the different materials showed that monocytes/macrophages on the 200 nm alumina surface secreted significantly higher levels of IL-1*β* than the cells attached to the 20 nm alumina membrane (Figure 3(a)). TNF-*α* secretion showed the same tendency (Figure 3(b)).

### 3.4. Scanning Electron Microscopy

**Figure 4:**
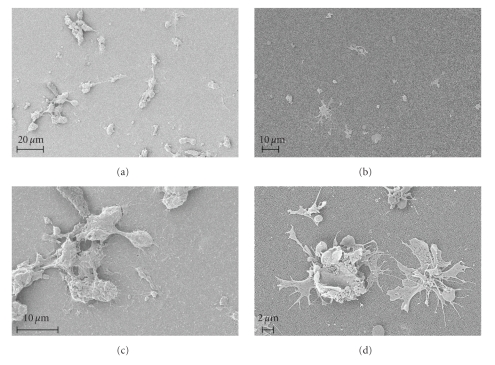
SEM micrographs of monocytes/macrophages after 24 h of incubation on nanoporous alumina. MM on the 200 nm-pore membranes ((b) and (d)) show clear signs of activation (rough plasmalemma and extended filipodia), while cells on the 20 nm-pore membranes ((a) and (c)) appear more round shaped with little membrane ruffling and no established filipodia extensions. A higher tendency toward cell clustering and fusion was also observed on the 20 nm-pore alumina (c).

SEM micrographs show that cell morphology is affected by the different surfaces (Figures 4(a)–4(d)). The 20 nm-pore alumina membranes induced lower degree of cell spreading (Figures 4(a) and 4(c)) as MM appeared round-shaped and less spread than the cells adhering on the 200 nm-pore membranes (Figures 4(b) and 4(d)). The 200 nm-pore surface presented cells with rough plasmalemma and extensive filopodia, both typical signs of activation (Figure 4(d)). Cells on the 20 nm-pore alumina, although showing some degree of membrane ruffling, did not show well-established filipodia extensions, thus indicating a lower degree of activation (Figure 4(c)). Furthermore, the cells contacting this type of porous alumina seem to form clusters and fuse into giant cells (Figure 4(c)). Conversely, the relatively fewer cells observed on the surface of the 200 nm-pore alumina were mostly adhering as single and well-spread cells with only a few cell aggregates being visible (Figure 4(d)). SEM studies also confirmed higher cell adhesion on 20 nm-pore alumina as compared to the 200 nm membrane (Figures 4(a) and 4(b)).

## 4. Discussion

The recent advance in engineering nanosize materials has shown the potential of controlling cell behavior and tissue/implant integration by nanotopography [12, 14, 25]. Previous works have demonstrated that nanoporous alumina can have an effect on neutrophil activation, complement activation, and platelet adhesion and activation [19, 22–24]. The effect of the surface nanotopography of several biomaterials on MM behavior has been widely described [26–31]. Hence, with the intention of getting a more complete understanding of the effect of nanoporosity on the inflammatory system we have chosen to evaluate the response of MM to alumina membranes with different nanoporosities. The results of this work have shown that the nanoporesize of the studied material affects both MM adhesion and activation. The different activation states were reflected by morphological changes and secretion levels of proinflammatory cytokines. 

A unique pattern of cell adhesion was observed on the 20 nm-pore alumina, where the cells seem to prefer the formation of clustering rather than spreading on the surface. Clustering is the typical behavior of cells when in contact with substrates unable to promote cell adhesion. In the case of the 20-nm porous alumina substrates, cells showed a tendency to form clustering, and they established only limited connection with the substrate thus suggesting that this material can limit monocytes/macrophages adhesion and activation. The mild activation of these adhering MM was confirmed by the analysis of proinflammatory cytokines TNF-*α* and IL-1*β*. Conversely MM adhering on the 200 nm-pore alumina membranes were able to establish a relatively strong contact with the surface and were activated at levels significantly higher than MM adhering on the 20 nm-pore membranes.

The cytokine normalized data provides information regarding the effect of the different nanoporosities on single cells and indicates that 200 nm pore size surface promotes higher MM activation. 

For a more clinically relevant approach, attention should be paid to the total levels of cytokines. When evaluating total levels of cytokines, higher levels of IL-1*β* were found for MM culture on 200 nm alumina compared with cells on 20 nm membranes: 20 nm (10 ± 1.0) 10^3^ pg/mL, 200 nm (13 ± 0.7) 10^3^ pg/mL, *P* = .100. Although this difference is not significant, a tendency can be established, indicating that 20 nm alumina most probably will lead to a lower overall inflammatory response as compared to the 200 nm membranes. 

LDH activity was measured to determine if cytokine release was caused by the loss of cell integrity as a consequence of different cytotoxic effects by the biomaterials. The lack of any significant difference between the LDH release of the cells adhering on alumina-based surface and on Thermanox suggested that the release of enzymatic activity can be attributed to the degranulation process that the cells are likely to undergo during their relative activation by surface contact. Indeed, these levels were ten times lower than those found in the positive controls, where cell membrane integrity was deliberately disrupted by surfactant treatment. These differences suggest lack of cytotoxicity by the materials and corroborate the hypothesis of an active release of cytokines by the activated cells.

Two main mechanisms can be proposed to explain how surface topography may affect MM adhesion and activation. Firstly, it is well established that material nanotopography affects protein adsorption, not only in terms of amount but also in dictating conformational changes, orientation, and exposure of cell-binding sites [7, 32]. Secondly, surface topography has been found to affect spreading, proliferation and differentiation of cells [15, 25, 33]. It has been proposed that cells are capable of sensing nanometric structures and thereby of responding to these features by changing the spatial organization of their cytoskeleton through membrane receptors. These variations are known to have an effect on gene expression and, as a consequence, on cell phenotype and functions [25]. 

From the numerous studies thus far published it is very difficult to single out the effects of adsorbed proteins on MM adhesion and activation [34, 35]. In vitro studies present a wide spectrum of materials, where chemistry and topography have not always been varied in a systematic and truly comparable manner. In addition, there is a great variation in the use of proteins as well as their concentrations and the conditions for the adsorption processes [36–42]. There is a consensus about the role of fibrinogen and C3 in promoting MM adhesion to biomaterial surfaces [43, 44]. However, the effect of adsorbed proteins on cytokine release varies between different studies. For instance, Shen et al. [39] describe that fibrinogen adsorbed on several polymers surfaces does not promote TNF-*α* release. The authors propose that monocyte integrin receptors participate mostly in cell adhesion, rather than directly activating MM for cytokine release. On the contrary, Gretzer [45] describes a fibrinogen-mediated proinflammatory effect with increased monocyte secretion of IL-1*β* and TNF-*α* and decreased secretion of IL-10. Interactions of adsorbed IgG with monocyte Fc receptors are also documented to promote the release of IL-1*β* and TNF-*α* by adherent monocytes [46, 47]. Jenney and Anderson [37] proposed that preadsorbed IgG promotes long-term macrophage adhesion, an effect that is mediated by Fab and *F*(*ab*′)_2_ fragments instead of the Fc fragment. 

A previous work has demonstrated that 200 nm-pore alumina adsorbed significantly higher amounts of the plasma proteins IgM, IgG, C3, and C1q than the 20 nm-pore membranes [24]. Thus, the results on MM adhesion presented in this paper prove that not only the amount of adsorbed protein but also the protein conformation and orientation may play an important role for cellular adhesion. Furthermore, MM could “sense” the topography and as a result might expose different membrane receptors [25, 26]. Hence, the reduced number of adherent cells on the 200 nm-pore alumina could be related to changes in protein conformation and/or MM “sensing” the different nanometric structure, thus leading to a less favorable exposure of key binding receptors. Instead, MM seem to adopt a configuration favorable for exposure of activation receptor-binding sites (e.g., receptors for Fc), and as a result, they become highly activated, thus producing high levels of cytokines.

Concerning the increased cell attachment on the 20 nm-pore alumina, it may be speculated that in addition to MM exposing adhesion receptor-binding sites, the cells express receptors favouring giant cell formation. However, despite the relatively higher cell number, the cytokine release on this type of membrane is less pronounced than on the 200 nm-pore membranes. Hence, as proposed by Young el al. [47], it can be hypothesized that greater activation energy is required to activate the receptors of MM attached to the 20 nm-pore surface as compared to the 200 nm-pore alumina.

C5a and C3a are anaphylatoxins generated when the complement systems are activated. C5a stimulates the release of inflammatory cytokines by MM [48]. C3a has also been described to enhance TNF-*α* and IL-1*β* synthesis by adherent monocytes [49]. As blood exposure to 200 nm-pore alumina leads to much higher complement activation than when exposed to the 20 nm-pore membranes [24], the increased levels of C5a and C3a could also contribute to the difference observed in cytokine secretion between the membranes. The mechanisms that regulate material-induced MM activation are not yet completely understood. However, in the case of nanoporous alumina, a direct relationship between complement activation and MM cytokine release can be postulated. 

The present work shows that by changing the nanopore size of alumina it is possible to modulate MM behavior. In addition, this data opens the possibility of exploiting some of the beneficial functions of macrophages in promoting tissue repair rather than chronic inflammation. Additional studies to determine the fate of the cells over longer period of time as well as the secretion of other growth factors are needed for further understanding of the effect of surface nanotopography on MM.

## 5. Conclusions

The present study shows that alumina nanotopography affects MM behavior. We found a clear difference in cell adhesion and activation between alumina membranes with 20 nm pores as compared to membranes with 200 nm pores. Few but highly activated cells adhered to the 200 nm membrane in contrast to many but less activated MM on the 20 nm surface. Regardless of the specific clinical applications of these types of surfaces, the data of this paper emphasizes the role of material nanotexture in dictating inflammatory cell responses and implies that nanotopography can be exploited to subtly control the inflammatory potential of medical implants.

## References

[1] Anderson JM (2001). Biological responses to materials. *Annual Review of Materials Science*.

[2] Anderson J, Ratner BD, Hoffman AS, Schoen FJ, Lemons JE (2004). Inflammation, wound healing and the foreign body response. *Biomaterials Science: An Introduction to Materials in Medicine*.

[3] Tang L, Eaton JW (1995). Inflammatory responses to biomaterials. *American Journal of Clinical Pathology*.

[4] Norde W (2008). My voyage of discovery to proteins in flatland ...and beyond. *Colloids and Surfaces B*.

[5] Wilson CJ, Clegg RE, Leavesley DI, Pearcy MJ (2005). Mediation of biomaterial-cell interactions by adsorbed proteins: a review. *Tissue Engineering*.

[6] Horbett TA (1993). Principles underlying the role of adsorbed plasma proteins in blood interactions with foreign materials. *Cardiovascular Pathology*.

[7] Kasemo B, Gold J (1999). Implant surfaces and interface processes. *Advances in Dental Research*.

[8] Wahlgren M, Arnebrant T (1991). Protein adsorption to solid surfaces. *Trends in Biotechnology*.

[9] Horbett T, Ratner BD, Hoffman AS, Schoen FJ, Lemons AS (2004). The role of adsorbed proteins in tissue response to biomaterials. *Biomaterials Science: An Introduction to Materials in Medicine*.

[10] Xia Z, Triffitt JT (2006). A review on macrophage responses to biomaterials. *Biomedical Materials*.

[11] Ratner BD, Bryant SJ (2004). Biomaterials: where we have been and where we are going. *Annual Review of Biomedical Engineering*.

[12] Yim EKF, Leong KW (2005). Significance of synthetic nanostructures in dictating cellular response. *Nanomedicine*.

[13] Tirrell M, Kokkoli E, Biesalski M (2002). The role of surface science in bioengineered materials. *Surface Science*.

[14] Goldberg M, Langer R, Jia X (2007). Nanostructured materials for applications in drug delivery and tissue engineering. *Journal of Biomaterials Science*.

[15] Flemming RG, Murphy CJ, Abrams GA, Goodman SL, Nealey PF (1999). Effects of synthetic micro- and nano-structured surfaces on cell behavior. *Biomaterials*.

[16] Price RL, Haberstroh KM, Webster TJ (2003). Enhanced functions of osteoblasts on nanostructed surfaces of carbon and alumina. *Medical and Biological Engineering and Computing*.

[17] Curtis ASG, Gadegaard N, Dalby MJ, Riehle MO, Wilkinson CDW, Aitchison G (2004). Cells react to nanoscale order and symmetry in their surroundings. *IEEE Transactions on Nanobioscience*.

[18] Nguyen KT, Shukla KP, Moctezuma M, Liping T (2007). Cellular and molecular responses of smooth muscle cells to surface nanotopography. *Journal of Nanoscience and Nanotechnology*.

[19] Karlsson M, Johansson A, Tang L, Boman M (2004). Nanoporous aluminum oxide affects neutrophil behaviour. *Microscopy Research and Technique*.

[20] Thormann A, Teuscher N, Pfannmöller M, Rothe U, Heilmann A (2007). Nanoporous aluminum oxide membranes for filtration and biofunctionalization. *Small*.

[21] Thompson GE (1997). Porous anodic alumina: fabrication, characterization and applications. *Thin Solid Films*.

[22] Ferraz N, Carlsson J, Hong J, Ott MK (2008). Influence of nanoporesize on platelet adhesion and activation. *Journal of Materials Science*.

[23] Ferraz N, Hong J, Karlsson Ott M (2010). Procoagulant behavior and platelet microparticle generation on nanoporous alumina. *Journal of Biomaterials Applications*.

[24] Ferraz N, Nilsson BO, Hong J, Ott MK (2008). Nanoporesize affects complement activation. *Journal of Biomedical Materials Research A*.

[25] Kriparamanan R, Aswath P, Zhou A, Tang L, Nguyen KT (2006). Nanotopography: cellular responses to nanostructured materials. *Journal of Nanoscience and Nanotechnology*.

[26] Wójciak-Stothard B, Curtis A, Monaghan W, Macdonald K, Wilkinson C (1996). Guidance and activation of murine macrophages by nanometric scale topography. *Experimental Cell Research*.

[27] Khang D, Liu-Snyder P, Pareta R, Lu J, Webster TJ (2009). Reduced responses of macrophages on nanometer surface features of altered alumina crystalline phases. *Acta Biomaterialia*.

[28] Kim J, Khang D, Jong EL, Webster TJ (2009). Decreased macrophage density on carbon nanotube patterns on polycarbonate urethane. *Journal of Biomedical Materials Research A*.

[29] Refai AK, Textor M, Brunette DM, Waterfield JD (2004). Effect of titanium surface topography on macrophage activation and secretion of proinflammatory cytokines and chemokines. *Journal of Biomedical Materials Research A*.

[30] Paul NE, Skazik C, Harwardt M (2008). Topographical control of human macrophages by a regularly microstructured polyvinylidene fluoride surface. *Biomaterials*.

[31] Hsu SH, Tang CM, Lin CC (2004). Biocompatibility of poly(e-caprolactone)/poly(ethylene glycol) diblock copolymers with nanophase separation. *Biomaterials*.

[32] Sutherland DS, Broberg M, Nygren H, Kasemo B (2001). Influence of nanoscale surface topography and chemistry on the functional behaviour of an adsorbed model macromolecule. *Macromolecular Bioscience*.

[33] Curtis A, Wilkinson C (1997). Topographical control of cells. *Biomaterials*.

[34] Thomsen P, Gretzer C (2001). Macrophage interactions with modified material surfaces. *Current Opinion in Solid State and Materials Science*.

[35] Kao WJ (1999). Evaluation of protein-modulated macrophage behavior on biomaterials: designing biomimetic materials for cellular engineering. *Biomaterials*.

[36] Kao WJ, Hubbell JA, Anderson JM (1999). Protein-mediated macrophage adhesion and activation on biomaterials: a model for modulating cell behavior. *Journal of Materials Science*.

[37] Jenney CR, Anderson JM (2000). Adsorbed IgG: a potent adhesive substrate for human macrophages. *Journal of Biomedical Materials Research*.

[38] Jenney CR, Anderson JM (2000). Adsorbed serum proteins responsible for surface dependent human macrophage behavior. *Journal of Biomedical Materials Research*.

[39] Shen M, Garcia I, Maier RV, Horbett TA (2004). Effects of adsorbed proteins and surface chemistry on foreign body giant cell formation, tumor necrosis factor alpha release and procoagulant activity of monocytes. *Journal of Biomedical Materials Research A*.

[40] Shen M, Horbett TA (2001). The effects of surface chemistry and adsorbed proteins on monocyte/macrophage adhesion to chemically modified polystyrene surfaces. *Journal of Biomedical Materials Research*.

[41] Bonfield TL, Colton E, Anderson JM (1989). Plasma protein adsorbed biomedical polymers: activation of human monocytes and induction of interleukin 1. *Journal of Biomedical Materials Research*.

[42] Bonfield TL, Colton E, Marchant RE, Anderson JM (1992). Cytokine and growth factor production by monocytes/macrophages on protein preadsorbed polymers. *Journal of Biomedical Materials Research*.

[43] Tang L, Ugarova TP, Plow EF, Eaton JW (1996). Molecular determinants of acute inflammatory responses to biomaterials. *Journal of Clinical Investigation*.

[44] Mcnally AK, Anderson JM (1994). Complement C3 participation in monocyte adhesion to different surfaces. *Proceedings of the National Academy of Sciences of the United States of America*.

[45] Gretzer C (2000). *Macrophage-material surface interactions*.

[46] Van De Winkel JGJ, Capel PJA (1993). Human IgG Fc receptor heterogeneity: molecular aspects and clinical implications. *Immunology Today*.

[47] Young TH, Lin DT, Chen LIY (2000). Human monocyte adhesion and activation on crystalline polymers with different morphology and wettability in vitro. *Journal of Biomedical Materials Research*.

[48] Nilsson B, Ekdahl KN, Mollnes TE, Lambris JD (2007). The role of complement in biomaterial-induced inflammation. *Molecular Immunology*.

[49] Takabayashi T, Vannier E, Clark BD (1996). A new biologic role for C3a and C3a desArg: regulation of TNF-*α* and IL-1*β* synthesis. *Journal of Immunology*.

